# RELATIONSHIP BETWEEN DONOR QUALITY AND RECIPIENT GRAVITY IN LIVER TRANSPLANT

**DOI:** 10.1590/0102-672020190001e1499

**Published:** 2020-07-08

**Authors:** Alexandre Coutinho Teixeira de FREITAS, Júlio Cezar Uili COELHO, Manoelle Risnei WATANABE, Rachel Lins das Chagas LIMA

**Affiliations:** 1 Department of Surgery, Federal University of Paraná, Curitiba, Paraná, Brazil; 2 Student, Medical School, Federal University of Paraná, Curitiba, Paraná, Brazil

**Keywords:** Liver transplantation, Donor selection, Waiting list, Transplante de fígado, Seleção do doador, Lista de espera

## Abstract

***Background*:**

Tools such as MELD score and DRI are currently used to predict risks and benefits on liver allocation for transplantation.

***Aim*:**

To evaluate the relation between donor quality and recipient severity on liver allocation.

***Methods*:**

Liver transplants performed in 2017 and 2018 were evaluated. Data were collected from Paraná’s State Government Registry. DRI was evaluated in relation to recipient MELD score and position on waiting list.

***Results*:**

It was observed relation between DRI and position on waiting list: higher risk organs were allocated to recipients with worse waiting list position. There was no relation between DRI and MELD score. Afrodescendents and elderly donor organs were allocated to lower MELD score and worse waiting list position recipients.

***Conclusion*:**

There is no relation between DRI and MELD on liver allocation. However, DRI interferes with allocation decision based on recipients waiting list position. Donor race and age interfere on both recipient MELD score and waiting list position

## RESUMO

***Racional*:**

Visando prever riscos e benefícios na alocação de órgãos no transplante hepático, são utilizados sistemas como o MELD, atual critério classificatório da lista de espera de transplantes, e o DRI, ferramenta que avalia fatores de risco do doador.

***Objetivo*:**

Relacionar a qualidade do doador de fígado com a gravidade do seu receptor.

***Métodos*:**

Foram avaliados os transplantes hepáticos realizados entre 2017 e 2018 no Estado do Paraná. Os dados foram coletados no sistema eletrônico da Central Estadual de Transplantes. Informações dos doadores foram relacionadas às dos receptores através da relação entre o DRI e o MELD e a posição do receptor na lista de espera.

***Resultados*:**

Foram avaliados 520 doadores e 520 receptores. Observou-se relação entre DRI e posição na lista de espera: órgãos de doadores com maior risco pelo DRI foram alocados para receptores com pior posição na lista de espera. Não houve correlação entre o DRI e o MELD. No entanto, órgãos de doadores da raça negra ou com idade maior ou igual a 60 anos foram alocados para receptores com MELD mais baixo e com pior posição na lista de espera.

***Conclusão*:**

Não existe relação entre o DRI e o MELD na distribuição de enxertos hepáticos no Estado do Paraná. O DRI interfere na alocação de acordo com a posição do receptor na lista de espera. A idade e a raça dos doadores interferem na alocação de acordo com o MELD e a posição em lista dos receptores.

## INTRODUCTION

Liver transplantation is the only curative therapeutic measure for patients with terminal liver disease. In Brazil, it is estimated that the annual need for livers for transplantation is approximately 5,000 organs. Nevertheless not even half of that amount is obtained. Of the 10,778 potential donors notified in 2018, only 3,531 became effective donors^13^. Faced with this organ shortage, one of the main challenges of liver transplantation is to optimize the allocation of organs between donors and recipients in order to maximize recipient survival and those who are still on the waiting list. Recently, several systems have been proposed in order to predict risks and benefits in graft allocation. They use variables from donors, recipients, or both^5^. 

MELD score (Model for End-Stage Liver Disease) is the most used worldwide. It stands out for its objectivity in predicting the mortality of patients with end-stage liver disease awaiting a transplant^8^. It is based on three widely available variables: serum bilirubin level, serum creatinine level and international standardized ratio (INR)^8^. Because of its effectiveness, in 2004 MELD was adopted as liver allocation criteria in the United States and in 2006 in Brazil^12^ ^,^ ^19^. 

Among the studies that proposed to evaluate the success of liver transplantation according to risk factors associated with the donor, the Donor Risk Index (DRI) is the most employed. It was idealized by Feng et al^9^. and was based on data from 20,023 transplants performed between 1998 and 2002 in the United States. Calculated from donor and transplant variables, its value must be interpreted as the relative risk of graft loss from a specific donor in relation to an “ideal” case. 

Presently, once an organ becomes available for transplantation, it is up to the team responsible for the patient who is first on the waiting list to accept or not that graft, according to what they consider to be the most beneficial. If denied, the liver is offered to the next person on the waiting list. Studies show that the factors that most interfere in this decision are those related to the quality attributed to the donor organ^11^. It is also observed that livers with higher DRI tend to be denied more frequently than those with lower DRI, being assigned to patients with worse positions on the waiting list, and consequently, lower MELD^9^. 

This strategy aims to guarantee the best possible prognosis for the patient with the greatest clinical severity, accepting the risks of a longer waiting time for an organ in the hope of obtaining a better one soon. It is questioned, however, whether the tendency to preserve critically ill patients from receiving organs with lower quality is in fact more effective in terms of overall survival benefit.

 Considering this organ distribution pattern observed internationally, added to the lack of studies that address DRI within the Brazilian liver transplant scenario, the objective of this study is to correlate the quality of the liver donor according to the severity of its recipient in the State of Paraná. 

## METHODS

The study was approved by the Research Ethics Committee of the Hospital das Clínicas, Universidade Federal do Paraná, under number 09045619.2.0000.0102, with the agreement of the Paraná State Transplant Agency and the Paraná State Health Department. 

Data from deceased donors and recipients of liver transplants performed from January2017 toDecember 2018 in the State of Paraná were used.The patients were registered at the Government Transplantation Agency.Data was obtained during the months of July and August 2019 through a computerized management system.

The following donor information was collected: gender, age, height, body mass index (BMI), race, cause of death, type of graft (split or whole liver), type of donation (deceased donor or donor after cardiac arrest), serum sodium, local or regional organ harvesting and cold ischemia time.The DRI was calculated according to the description byFenget al^9^.The sample was divided into two groups: low DRI and high DRI.The cutoff point between them was established by the value of the third quartile.

Concerning the recipients, the following data were collected: gender, age, cirrhosis etiology, creatinine, bilirubin, INR, serum sodium, listing as a priority and reason, listing as an emergency priority and reason, position on the waiting list at the time of transplant, transplantation center location, and MELD score.The MELD value obtained directly from the electronic system of the Government Transplantation Agency has already considered the score of priority situations described by Brazilian Government Ordinance No. 2,600 / 2009.The recipients were divided into three MELD categories: low (<15), intermediate (15-30) and high (> 30).

The following information regarding donors was related to the recipients MELD categories and position on the waiting list: DRI, age, race, BMI, local or regional organ harvesting and cause of death.

### Statistical analysis

Mann-Whitney andKruskal-Wallistests were used to elaborate associations between the researched data.The level of statistical significance was set at 5%.It was used the statistical software R (R Core Team, 2015) version 3.6.1.

## RESULTS

Data from 520 donors and 520 recipients were included.All liver transplants were performed in the state of Paraná in non-pediatric patients (over 12 years old) between January 2017 and December 2018.

Donor characteristics are shown in Table 1. Of the 520donors,314(60.4%) were male.The mean age was 42±16 years, with 75 (14.4%) aged 60 years and over.As for the body mass index, it was found that 279 (53.65%) patients were overweight or obese.The main causes of donor death were traumatic brain injury (n=176; 33.85%) and hemorrhagic stroke (n=164; 31.54%).It was found that 152 (29.23%) of the organs were obtained in the same metropolitan region where the transplant was performed, and 368 (70.77%) were obtained outside of the metropolitan region where the transplant was performed.The average DRI value was 1.54±0.21.The cut-off value of 1.6 was considered for the division between high and low DRI.In the low DRI group, the mean value was 1.47±0.09, and in the high DRI group, 1.78±0.28.

**TABLE 1:** Donor characteristics

Variables	LOW DRI	High DRI	TOTAL
Description	(<1.6)	(= 1.6)	
Donors	390 (75%)	130 (25%)	520 (100%)
Genre			
Male	284 (72.8%)	30 (23.08%)	314 (60.4%)
Feminine	106 (27.2%)	100 (76.9%)	206 (39.6%)
Age (years)	40.3 ± 14.7	46.9 ± 18.5	42 ± 16.0
Elderly (≥60 years)	39 (10%)	36 (27.7%)	75 (14.4%)
Height (cm)	172.6 ± 7.36	160.9 ± 7.1	169.7 ± 8.9
BMI			
Low weight	6 (1.54%)	5 (3.85%)	11 (2.12%)
Normal	169 (43.3%)	61 (46.92%)	230 (44.23%)
Overweight	177 (45.4%)	50 (38.46%)	227 (43.65%)
Grade 1 obesity	35 (8.97%)	12 (9.23%)	47 (9.04%)
Grade 2 obesity	3 (0.77%)	2 (1.53%)	5 (0.96%)
Grade 3 obesity	-	-	-
Donor race			
White	300 (76.92%)	92 (70.77%)	392 (75.38%)
Black	17 (4.36%)	17 (13.08%)	34 (6.54%)
Brown	72 (18.46%)	19 (14.62%)	91 (17.5%)
Yellow	1 (0.26%)	2 (1.54%)	3 (0.58%)
Cause of death			
TBI	169 (43.33%)	7 (5.38%)	176 (33.85%)
HS	115 (29.45%)	49 (37.69%)	164 (31.54%)
IS	41 (10.51%)	13 (10%)	54 (10.38%)
Others	65 (16.66%)	61 (46.92%)	126 (24.23%)
Serum sodium n (%) meanmEq/l			
Hyponatremia	32 (7.69%) 129.5 ± 5.4	10 (7.69%) 129.7 ± 3.8	42 (8.08%) 129.6 ± 5.0
Normal	157 (40.26%) 140.6 ± 3.1	46 (35.38%) 141.5 ± 2.6	203 (39.04%) 140.8 ± 3.0
Hypernatremia	201 (51.54%) 154.8 ± 10.5	74 (56.92%) 159.1 ± 31.4	275 (52.88%) 156 ± 18.6
Transplant location			
Curitiba	333 (85.38%)	111 (85.38%)	444 (85.38%)
Cascavel	57 (14.62%)	19 (14.62%)	76 (14.62%)
Organ harvesting			
Local	117 (30%)	35 (26.92%)	152 (29.23%)
Regional	273 (70%)	95 (73.08%)	368 (70.77%)
Cold ischemia time (minutes)	266.4 ± 100.5	290.9 ± 107.9	272.5 ± 102.9
Average DRI	1.47 ± 0.09	1.78 ± 0.28	1.54 ± 0.21

SD=standard deviation;cm=centimeters;BMI=body mass index;TBI=traumaticbrain injury;HS=hemorrhagic stroke;IS=ischemic

Table 2 presents data from the 520 recipients, which were divided into three groups: 26 (5%) belonged to low MELD (<15), 439 (84.4%) to intermediate MELD (15-30) and 55 (10.6%) to high MELD (> 30) group.Male gender represented 65.8% of the recipients.Alcoholic cirrhosis (n=148; 28.4%) was the most prevalent diagnosis, followed by viral cirrhosis (n=98; 18.8%) andcryptogeniccirrhosis(n=64;12.3%).Priority was registered in 97recipients(18.6%), andhepatocarcinomawithin Milan criteria was the main reason.Emergency prioritization occurred in 3.46%:retransplantationdue toprimary graft failure (n=10; 55.6%) and fulminant liver failure (n=8; 44.4%).Waiting list position at the time of the transplant averaged 1.1 (± 0.3) in the high MELD group, 4.5 (± 7.3) in the intermediate MELD and 18.2 (± 21.1) in the low MELD.

**TABLE 2:** Recipient characteristics

Variables description	LOW MELD	INTERMEDIATE MELD	HIGH MELD	TOTAL
	(<15)	(15-30)	(> 30)	
Recipients sex	26 (5%)	439 (84.4%)	55 (10.6%)	520 (100%)
Male	21 (80.77%)	286 (65.15%)	35 (63.64%)	342 (65.77%)
Female	5 (19.23%)	153 (34.85%)	20 (36.36%)	178 (34.23%)
Age at transplant (years)	57.2±8.8	53.4±11.4	47.7±16.4	53±12.1
Diagnosis				
Primary liver cancer	1 (3.85%)	36 (8.2%)	1 (1.82%)	38 (7.31%)
Cirrhosis by HBV or HCV	10 (38.46%)	84 (19.13%)	4 (7.27%)	98 (18.85%)
Autoimmune hepatitis	1 (3.85%)	22 (5.01%)	7 (12.73%)	30 (5.77%)
Cryptogeniccirrhosis	1 (3.85%)	55 (12.53%)	8 (14.55%)	64 (12.31%)
Non-alcoholic fatty liverdisease	1 (3.85%)	47 (10.71%)	8 (14.55%)	56 (10.77%)
Metabolic diseases	-	1 (0.23%)	-	1 (0.19%)
Primary biliary cirrhosis	-	13 (2.96%)	-	13 (2.5%)
Secondary biliary cirrhosis	1 (3.85%)	6 (1.36%)	-	7 (1.35%)
Alcoholic cirrhosis	10 (38.46%)	127 (28.93%)	11 (20%)	148 (28.46%)
Refractory ascites	-	4 (0.91%)	-	4 (0.77%)
Fulminant hepatitis	-	2 (0.46%)	11 (20%)	13 (2.5%)
Hemochromatosis	-	4 (0.91%)	1 (1.82%)	5 (0.96%)
Neurological tumor liver metastases	-	2 (0.46%)	-	2 (0.38%)
Wilson’s disease	-	1 (0.23%)	2 (3.64%)	3 (0.58%)
Biliarytractatresia	-	2 (0.46%)	-	2 (0.38%)
Hepatopulmonarysyndrome	-	1 (0.23%)	-	1 (0.19%)
Multiple hepaticadenomatosis	-	1 (0.23%)	-	1 (0.19%)
Primary sclerosing cholangitis	-	6 (1.37%)	-	6 (1.15%)
Budd-Chiari syndrome	-	2 (0.46%)	-	2 (0.38%)
Others	1 (3.85%)	23 (5.24%)	2 (3.64%)	26 (5%)
Creatinine (mg/dl)	1.0±0.3	1.4±0.9	2.2±1.2	1.4±1.0
Bilirubin (mg/dl)	2.3±2.2	5.1±6.6	16.8±13.1	6.2±8.3
RNI	1.4±0.4	1.8±0.6	4.1±2.9	2±1.3
Recipient sodium n (%) meanmEq/ L				
Hyponatremia	4 (15.4%) 131.8±1.7	98 (22.3%) 130.7±3.2	14 (25.4%) 131.2±2.5	116 (22.3%) 130.8±3.1
Normal	19 (73.1%) 139.5±2.9	292 (66.5%) 138.8±2.6	28 (51%) 137.8±2.5	339 (65.2%) 138.8±2.6
Hypernatremia	3 (11.5%) 141.2±6.1	49 (11.2%) 138.8±6.6	13 (23.6%) 137.9±7.4	65 (12.5%) 138.7±6.7
Priority				
Yes	-	96 (21.87%)	1 (1.82%)	97 (18.65%)
No	26 (6.1%)	343 (78.13%)	54 (98.18%)	423 (81.65%)
Priority reason				
Hepatocarcinoma- Milan criteria	-	61 (63.54%)	1 (100%)	62 (63.9%)
SNT authorized	-	15 (15.63%)	-	15 (15.46%)
Hepatocarcinoma-downstaging	-	15 (15.63%)	-	15 (15.46%)
Adenomatosis	-	2 (2.08%)	-	2 (2.06%)
Hemangioma	-	1 (1.04%)	-	1 (1.03%)
Unresectablemetastatic neuroendocrine tumor	-	1 (1.04%)	-	1 (1.03%)
Late hepatic artery thrombosis	-	1 (1.04%)	-	1 (1.03%)
MELD score	12.4±2.1	21.7±3.4	39.4±10.8	23.1±7.6
Sodium MELD score	14.2±2.6	23.3±3.9	39.6±9.2	24.6±7.2
List position	18.2±21.1	4.5±7.3	1.1±0.3	4.8±8.8
Emergency priority	1 (3.85%)	7 (1.59%)	10 (18.18%)	18 (3.46%)
Retransplantationfor primary non-functioning	1 (100%)	5 (71.43%)	4 (40%)	10 (55.56%)
Fulminant liver failure	-	2 (28.57%)	6 (60%)	8 (44.44%)

HBV=hepatitis B virus;HCV=hepatitis C virus;SNT=National Transplant System

Table 3 shows the MELD score in the three groups according to the DRI.MELD values ​​of patients who received organs with low DRI and high DRI were 23.35±7.83 and 22.52±6.82, respectively, with no statistically significant difference between the two groups (p=0, 31).

**TABLE 3:** MELD score according to DRI

MELD	Low DRI (<1.61)	High DRI (= 1.61)	TOTAL	p **
Low MELD (<15)	12.5±1.92	12.13±2.64	12.38±2.12	0.908
Intermediate MELD (15-30)	21.77±3.38	21.66±3.57	21.74±3.42	0.701
High MELD (> 30)	39.55±11.41	38.82±8.32	39.4±10.8	0.957
TOTAL	23.35±7.83	22.52±6.82	23.14±7.6	0.319

** Mann-Whitney test

Table 4 shows organ distribution according to MELD category.In low DRI group, 18 (4.62%) organs were allocated to patients with low MELD, 328 (84.1%) to patients with intermediate MELD and 44 (11.28%) to patients with high MELD.In high DRI group, 8 (6.15%)organswere allocated to patients with low MELD, 111 (85.38%) to patients with intermediate MELD and 11 (8.46%) to patients with high MELD.

It was found that patients who received organs from low DRI donors had waiting list position of 4.57 ± 8.82, and those who received organs from high DRI donors, 5.55±8.53 (p=0.0435).

**TABLE 4:** Distribution of organs with low and high DRI according to the MELD category

DRI	Recipients			
	Low MELD (<15)	Intermediate MELD (15-30)	High MELD (> 30)	TOTAL
Low DRI (<1.61)	18 (4.62%)	328 (84.1%)	44 (11.28%)	390
High DRI (=1.61)	8 (6.15%)	111 (85.38%)	11 (8.46%)	130
TOTAL	26	439	55	520

MELD score of patients who received livers from donors under 60 years of age (23.35±7.26) was higher than the MELD of patients who received livers from donors of 60 years of age and over (21.9±9, 3, p=0.012).Livers from donors under the age of 60 were allocated to patients with 4.3±7.8 waiting list position, while those from donors of 60 years and over were allocated to patients with an average position of 7.9±12.7 (p=0.02749).Organs from black donors were allocated to patients with lower MELD score (20.76±4.88, p=0.042)compared to MELD score of the other groups.These and the other relationships between donors and recipients are shown in Table 5.

**TABLE 5:** List of donor characteristics with MELD and the position on the recipient’s waiting list

Donor variable	MELD		Waiting list position	
	Mean and standard deviation	p	Mean and standard deviation	p
BMI		p = 0.00247 *		p = 0.06738 *
Low weight	23.54±11.67		13.36±27.04	
Normal	23.85±7.20		4.14±7.85	
Overweight	22.65±7.93		5.11±8.4	
Grade 1 obesity	22.57±6.73		4.47±5.35	
Grade 2 obesity	17.4±3.91		7.2±4.92	
Harvesing		p = 0.2899 **		p = 0.355 **
Local	22.56±6.51		4.68±8.50	
Regional	23.29±8.00		4.88±8.87	
Age		p = 0.01263 **		p = 0.02749 **
<60 years	23.35±7.26		4.30±7.79	
≥ 60 years	21.91±9.3		7.92±12.74	
Race		p = 0.0042 *		p = 0.2232 *
White	22.92±7.34		5.16±9.55	
Black	20.76±4.88		5.38±7.21	
Brown	25.07±9.14		3.23±4.78	
Yellow	21.67±2.08		2.33±0.57	
Cause of death		p = 0.4092 *		p = 0.3281 *
TBI	23.81±82		4.59±9.56	
HS	22.53±6.29		4.96±9.18	
IS	21.93±6.53		5.04±7.09	
Others	23.53±8.58		4.86±7.69	

BMI=body mass index;TBI=traumatic brain injury;HS=hemorrhagic stroke;IS=ischemic stroke;*=Kruskal-Wallistest;**= Mann-Whitney test

Of 520 transplants, 217 were performed on patients who were at the top of the waiting list.Of the 390 organs with low DRI that were offered to patients at the top of the list, 170 (43.6%) were accepted;of the 130 organs with high DRI, 47 (36.2%) were accepted (Table 6).

**TABLE 6:** Relationship of the DRI with the number and MELD score of patients at the top of the waiting list

Description	Number of patients at the top of the waiting list	MELD of patients at the top of the waiting list	P **
Low DRI (<1.61)	170 (43.6%)	27.3±9.6	0.968
High DRI (= 1.61)	47 (36.2%)	26.6±8.7	
TOTAL	217 (41.7%)	27.1±9.4	

**=Mann-Whitney test

## DISCUSSION

In this study, DRI values ​​were homogeneous.In this score, three variables have the strongest association with donor risk:age over 60 years, donation after cardiac arrest andgraftpartition^10^.In this study, only the first variable was present on DRI calculation, since in Brazil donation is not allowed after cardiac death and no liver partition was observed in the sample.

The relative uniformity of DRI was also evident on organ quality versus recipient disease severity analysis. It was noticed that, within each range of MELD score, its value had no significant variation between high and low DRI groups.A possible explanation is that the homogeneity of DRI is directly associated with the donor sample homogeneity in the study.

According to data from the Brazilian Association of Organ Transplantation (ABTO), referring to the year 2018, Paraná was the Brazilian state with the largest relative number of effective donors, which corresponds to 47.7 per million inhabitants, while the average in Brazil is only 17 effective donors per million^6^.The rate in Paraná is comparable to that in Spain (46.9 donors/million), a country that stands out internationally due to the high rate of organ procurement^13^.It can be inferred that with a more comprehensive organ offer the selection of grafts can be more rigorous as to its quality. It does not happen in places like the United States, Europe and several states of Brazil, in which a greater imbalance between supply and demand requires acceptance of marginal donors^14^ ^,^ ^18^.

The donors average age was 42 years, which is close to that which would be present as an ideal donor byFengetal^9^.The rate of donors aged 60 years old and over corresponded to 14.4% of the total.This finding is inferior to that found in other studies. Bloket al. identified 25% of elderly donors^3^.In the high DRI group, the elderly population represented 27.7% of donors, while in the low DRI was 10%.Regardless the DRI, it was documented that organs of elderly patients were distributed to recipients with lower MELD score and worse waiting list position.This demonstrates a greater rejection of these organs by the teams responsible for the patients that occupied the top places on the waiting list, probably due to the greater risk when using livers from elderly donors^1^.

Similarly, organs of black donors were distributed to recipients with lower MELD score.Studies show that transplantation from black donors to white patients has a higher risk of failure (up to 27.4%) and also a lower survival^16^.

Despite the characteristics that attribute increased risk to a graft are already known by transplantation teams, this knowledge does not eliminate the complexity involved in choosing to accept or deny an organ when it is offered.In addition to the quality of the donated organ, it is necessary to consider the recipient’s clinical condition at the time of the offer, the likelihood of deterioration of his condition, the potential for short and long-term success with the transplant, the age of the recipient, among others.

In the present study, 41.7% of the organs were accepted immediately for patients who were at the top of the waiting list.The acceptance rate was higher in low DRI donor group than in high DRI, with values ​​of 43.6% and 36.2%, respectively.A study that analyzed OPTN (Organ Procurement and TransplantationNetwork) data for more than twenty thousand transplants showed a similar acceptance rate, corresponding to 37.4%^7^.

One study evaluated the reasons given for organ refusal offered to top placed waiting list candidates^11^.It found that 68% of rejected organs were justified by the quality and age of the donor and 15% by other factors^11^.Thus, the importance of donor risk factors in organ selection by transplantation teams is reinforced.

The consequence of valuing the quality of the donated organ is the tendency to allocate organs with more risk factors to patients with less severity, which has been reported in the literature and was observed in the present study^4^ ^,^ ^17^. Donors with higher risk were allocated to patients on the worst waiting list position.The problem associated with this situation is based on two issues.The first is that by denying an organ to a seriously ill patient, his life is at risk, as there is no guarantee that he will survive until the offer of a more favorable organ.Laiet al. showed that 84% of patients who died or were removed from the waiting list were offered at least one liver^17^. On the other hand, there is an even ethical dilemma in accepting organs of inferior quality than the one that the patient has the potential to receive.Although the relative benefit in survival may be different when comparing different MELD categories, the donor’s characteristics still have a significant impact, to a greater or lesser degree, on the outcome of the transplant.

## CONCLUSION

There is no relationship between donor’s risk assessed by DRI and recipient’s MELD score in liver grafts allocation in the State of Paraná.However, organs at greatest risk by DRI are allocated to patients in worst position on the waiting list.Organs from elderly and black donors are also allocated to patients in worst position on the waiting list and with lower MELD score.
